# Exercise

**Published:** 1884-02

**Authors:** 


### EXERCISE,

Like cold water, is an excellent thing in its place, and out of its place, may be mischievous, deadly. A man in a chill may be chilled to death by being soused into cold water, but, if he survives it, it may cure him. It is too dangerous a remedy unless under intelligent direction. A man on the verge of cholera will infallibly fall into its most fatal form, if he keeps on his feet, this is as certain, as that an unsupported stone will fall to the earth.

*The benefit of Exercise consists in knowing the* HOW *and the* WHEN.

In a previous number of the Journal, the question of the HOW, was fully answered—the WHEN, we propose to discuss at this time, with reference to those whose business is of a sedentary character, whose general occupation is not one of bodily activity, of those more especially still, whose main employment is head work, the banker, the scholar, the artist, the clergyman, the clerk, the book-keeper, &c.

It is a conceded fact, that after a night of sound repose, a man wakes up, feeling that he is invigorated, he has a certain amount of nervous energy to be expended during the subsequent day, a part through the brain and a part through the body; and having been busy, he feels at nightfall weary and tired.

Any man must see, that he can work best in the morning, the strokes then count double, physically speaking. On the same principle, the brain works best in the morning, being backed, as it is, by a large supply of nervous power. But suppose a man makes his living by head work, suppose his occupation is of such a character, that success depends on great nicety of observation and clearness of judgment, and strength of combinations, it is apparent that the larger amount of nervous energy undrawn upon, the more efficiently he can work. But suppose that supply has been taxed by a long walk, or one or two hours of bodily labor, the brain is that much crippled for want of resources, the *deposite* is just that much reduced, there are just that much less means to "operate" upon. Therefore,

Ye Wall-street men! ye brain-workers! do not walk down town to your business of a morning, "*to settle your breakfast,*" as it is expressed. The best settler of a meal is bodily quietude and mental hilarity, a joyous laugh on the sofa amid your family. No horseback traveller will get into his saddle the moment his noble animal has fed. Of two dogs, eating heartily one left in his manger, the other taken on a hunt, after several hours, both were killed, the one at rest had passed all the food from his stomach, while, in that of the other it was almost unchanged, from the time of its having been eaten.

The time for physical exercise to the brain-worker is in the after part of the day. When the brain is wearied with work, the whole body feels tired. But who has not found, that under such circumstances, a leisure walk or ride, or light work, invigorates, refreshes both body and mind. The other morning, as we were riding down Broadway, we saw a noted banker, staving a-head with all his might, as if he were walking for a wager against time. His body was on the side-walk, the other part of him was in his counting-room. He saw nobody, spoke to nobody; his eyes were bent on the pavement; his head was far forward; his whole body made the segment of a circle; he was consuming his nervous power at a fearful rate; his candle was burning at both ends; all his energy was going out at his heels and head; while the stomach, which should have monopolized the supply, until the breakfast had been taken care of, was left neglected. Now, can any man of reflection imagine that our eye was fixed upon a stout, robust looking person, whose firm tread, and keen eye and portly mien, and satisfied and composed expression of countenance—all bespoke the man? No! No! He was a little bit of a thin bodied, weazen-faced, care-worn, ricketty-treaded individual, that a very moderate puff of wind might have swept into the gutter; his face all wrinkles, the corners of his mouth turned down, with a countenance so distressingly anxious and solemcholy, that even at this moment, we think of him with commiseration, and we wouldn't exchange our health for his hundreds of thousands. How strongly we believe in the doctrine of compensations!

Another item we must not overlook, as it has proved the death of many a man. Such a walk of a summer's morning, leaves a man in a perspiration; in that state he enters his office, which most generally feels cool to him, he pulls off his hat, and most probably changes his coat, and puts on his slippers, all of which being colder than the articles of dress just removed, cool off his body rapidly, and very often, before he is aware of it, he feels a little chilly, or at least, a little cooler than is comfortable; the reaction of this, is "fever." A single occurrence of this kind may be comparatively trifling, but, like a drop to the ocean, it is still felt; it does an appreciable harm, and being habitually repeated, it works in the course of years, the ruin of the constitution.

Be assured, reader, that it is attention to these apparently trifling things, which secures a long life of vigorous health· while their neglect has attached to it an unfailing penalty, the penalty of premature death or a life of daily aches and pains and symptoms, which, in the aggregate, are worse than being hung at once. Now, is it wise in you to dismiss the subject by saying, "Well! if I am to be everlastingly bothered with attention to such a multitude of little things, I may as well die off at the outset." Men do not reason thus in their efforts to accumulate money. Our most successful men, our millionaires, are men, who, from early life to the present hour, count up all the quarters of cents, pick up all the pins and save all the buttons and pieces of strings they come across; nor do they consider it a burden to do such things, on the contrary, it is an actual pleasure, and having got into the habit of it, it is no trouble at all. Thus it is with men who have the intelligence to see the importance of taking care of their health, the wisdom to know how it ought to be done, and the firmness of character to carry it out; it is pleasurable, because profitable, and they find it is even easier to do right than it is to do wrong, when one has got into the habit of it.

